# Intra-lesional Corticosteroids Versus Platelet-Rich Plasma Versus Platelet-Rich Fibrin for the Treatment of Oral Lichen Planus: A Systematic Review and Network Meta-Analysis

**DOI:** 10.7759/cureus.69973

**Published:** 2024-09-23

**Authors:** Khaled A Alshemmari, Saud Alzafiri, Mohammad Alajmi, Abdulaziz Alshammari, Sayed Hashem, Khaled Alzafiri, Reem AlQusaimi, Saleh Alajmi, Mohammd Aladwani, Gheith O Rasheed

**Affiliations:** 1 General Dentistry, Ministry of Health, Kuwait City, KWT; 2 Medicine and Surgery, Farwaniya Hospital, Ministry of Health, Kuwait City, KWT; 3 Dermatology, As'ad Al-Hamad Dermatology Center, Salwa, KWT; 4 Medicine and Surgery, Kuwait Institute for Medical Specializations, Kuwait City, KWT; 5 Dermatology, Farwaniya Hospital, Kuwait City, KWT; 6 Medicine and Surgery, The Hashemite University, Zarqa, JOR

**Keywords:** meta-analysis, oral lichen planus, corticosteroids, prf, prp

## Abstract

Oral lichen planus (OLP) is a potentially malignant disorder affecting the oral mucosa. Platelet concentrates, including platelet-rich plasma (PRP) and platelet-rich fibrin (PRF), have emerged as promising alternative treatments to corticosteroids. This study aims to comprehensively evaluate the effectiveness of PRP and PRF in the management of patients with OLP. We conducted a comprehensive search of PubMed, Scopus, Web of Science, and the Cochrane Library for randomized controlled trials (RCTs) involving patients with OLP comparing intralesional PRP or PRF with corticosteroids up to August 2024. The primary outcomes assessed were changes in lesion size, pain scores, and Thongprasom scores. Network meta-analysis (NMA) was used. Data were pooled using summary effect sizes with corresponding 95% confidence intervals (CIs) in a random-effects model based on the DerSimonian-Laird method. Eight studies comprising 157 patients and 250 lesions were included in the final analysis. Compared to corticosteroids, no significant differences were observed among PRF and PRP in terms of changes in lesion size, pain scores, clinical severity scores, and adverse events. NMA ranking showed that PRF was the best-ranking treatment in reducing lesion sizes (SUCRA values: 72.6%, 75.8%, 66.2%, 80.8%, and 77.5% at first, second, third, fourth, and eighth weeks of assessment), followed by corticosteroids, and PRP. Moreover, PRF was the best-ranking treatment in reducing pain score at the first, third, and eighth weeks of assessment (SUCRA values: 91.8%, 86%, and 85.9%), while PRP was the best intervention at the second and fourth weeks of assessment (SUCRA values: 61.3%, and 90.2%). Also, PRF was the best intervention in terms of Thongprasom scores at eight weeks of assessment (SUCRA value: 77.3%), while PRP was the best intervention at the fourth week of assessment with value of 78.1%. PRF and PRP showed comparable results with intralesional corticosteroids in all studied parameters. Considering treatments ranking, PRF was the best intervention. The optimal treatment modality for OLP varies on different clinical conditions.

## Introduction and background

Lichen planus (LP) is not a well-known major inflammatory condition [1]. Oral LP (OLP) is a type of LP that affects mucous membranes inside the mouth, genital, scalp, and nails [1-3]. OLP is an idiopathic disease and could be caused by an autoimmune-mediated cytotoxic T-cell response targeting keratinocytes, potentially triggered by genetic factors, infections (notably hepatitis C), medications, or environmental exposures [1, 2].

The diagnosis of OLP is challenging as various diseases may appear similar to OLP (i.e., contact stomatitis, squamous cell carcinoma, oral candidiasis, oral HPV) [2, 4]. It's important to understand how the disease works, how it shows up, and how to diagnose it in order to give the right treatment [5]. Patients with OLP frequently complain of dry mouth, and other symptoms such as burning sensation, pain, or discomfort, which increases depending on sensitivity to certain food [6, 7].

Treatment of OLP focuses on relieving the symptoms and managing and reducing the inflammation [2]. The topical application of steroids is the first line [2]. Moving forward, systemic corticosteroids and intralesional corticosteroids are used in different clinical settings in OLP [8]. Due to these lines side effects, and the growing field of autologous platelets concentrates [9], intentionally, new studies emerged platelet-rich fibrin (PRF) and platelet-rich plasma (PRP) as a potential line of treating OLP.

In this systematic review and network meta-analysis (NMA), we aimed to comprehensively assess the effectiveness of autologous platelets concentrates (PRP and PRF) in patients with OLP compared to intralesional corticosteroids providing the latest significant evidence for healthcare providers in this clinical setting.

## Review

Methods

This network meta-analysis was performed in accordance with the Preferred Reporting Items for Systematic Reviews and Meta-Analyses (PRISMA) statement for the NMA model [10] and followed the guidelines of NMA methodology delineated in the Cochrane Handbook for Systematic Reviews and Meta-Analyses [11].

Search Strategy and Data Sources

We comprehensively retrieved PubMed, Scopus, Web of Science, and Cochrane Library from inception till August 2024 without any language restrictions, with the following search strings: "platelet-rich plasma", "thrombocyte-rich plasma", "platelet-rich fibrin", "thrombocyte-rich fibrin", "corticosteroids", "oral lichen planus". Moreover, we adopted the snowball method for retrieving relevant articles from included RCTs and past meta-analyses to ensure comprehensive coverage of all related articles.

Eligibility Criteria and Study Selection

We included all randomized controlled trials (RCTs) with the following criteria: 1) patients with OLP; 2) intralesional injection of PRP or PRF as an experimental arm; 3) intralesional corticosteroids as a control arm; and 3) the outcomes studies were changes in lesion size, pain scores, and Thongprasom-scale scores. We excluded studies whose participants had systematic diseases, such as DM, autoimmune diseases, or hematological disorders; moreover, patients taking oral corticosteroids were excluded. Additionally, we excluded reviews, conference abstracts, book chapters, and unpublished data from the study.

Two authors independently performed the title, abstract, and full-text screening, and the selection of the final included studies were disputed with discussions. Any conflict during the screening phase was resolved via consensus with a third author.

Endpoints

The outcomes of interest were changes in the lesions size in cm, changes in the pain scores evaluated by the numeric rating scale (NRS) or visual analog scale (VAS), and changes in clinical scores evaluated by the Thongprasom-scale score.

For pain assessment, VAS and NRS are validated to be used as pain measurements for acute and chronic pain conditions [12]. VAS scale is a rating scale from 0 to 10, where "0" indicates no pain and "10" indicates the worst pain ever. NRS is similar to VAS for pain rating with a 10-scale score, where "0" indicates no pain and "10" indicates the most severe pain [13]. For clinical scores, Thongprasom-scale score was adopted to evaluate the size and shape of OLP lesions which varied from 0 to 5, where score 0 indicates normal mucosa; score 1 indicates a lesion with white stria, score 2 indicates a lesion with white stria and atrophic areas less than 1 cm^2^, score 3 indicates a lesion with white stria and atrophic areas larger than 1 cm^2^, score 4 indicates a lesion with white stria and erosive areas less than 1 cm^2^, and score 5 indicates a lesion with white stria and erosive areas larger than 1 cm^2^ [14].

Quality Assessment and Data Extraction

The Cochrane risk-of-bias tool-2 (ROB2) for RCTs [15] was employed by two independent authors to assess the risk of bias. The ROB-2 domains were randomization, deviations, missing data, outcome measurement, result selection, and other biases. Judgments were categorized as "low risk," "high risk," or "some concerns," with discrepancies resolved by a third author through consensus.

We extracted data from the included studies and summarized the evidence. The data consisted of baseline characteristics of the included patients, characteristics of included studies, and the main outcomes of the included studies using a designed Excel sheet tables (Microsoft, Redmond, Washington). Data on lesion size reported in mm^2^ was converted to cm^2^ all over the studies.

Statistical Analysis

We performed a frequentist approach for this network meta-analysis using the "network" package in STATA 18MP, which incorporates the direct and indirect estimates of different treatment arms to utilize a comprehensive comparison. Pooled data presented as mean difference (MD) and its 95% confidence interval (CI; heterogeneity was assessed using Cochrane's Q test or an I2 value ≥50% with a p-value of ≤0.05 was considered significant heterogeneity of which the random-effect model was declared. Inconsistency was detected using the Cochrane equation for heterogeneity if I2 value ≥50% with a p-value of ≤0.05 [16].

The network meta-analysis results were displayed as net league tables, and network plots were generated to visualize the comparison between interventions. Moreover, the surface under the cumulative ranking curve (SUCRA) approach was used to measure the overall ranking and presents a single number with each treatment arm. SUCRA values range from 0 to 100%. The higher the values of SUCRA, the higher the likelihood that the treatment is in the top rank [17]. We also used the adjusted funnel plots to explore the publication bias in our study [18].

Results

Search Results

**Figure 1:**
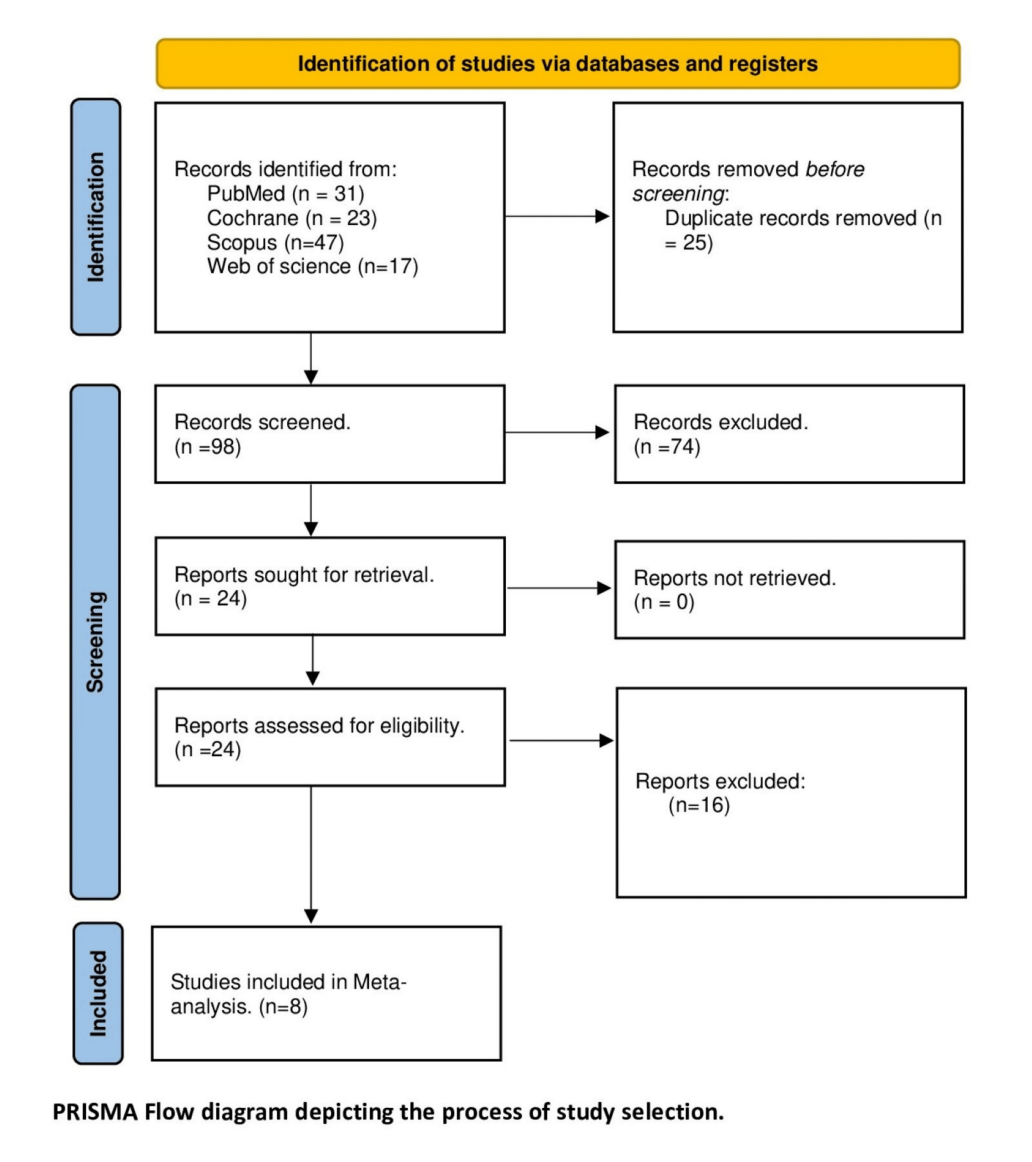
PRISMA flow diagram PRISMA - Preferred Reporting Items for Systematic Reviews and Meta-Analyses

Our search strategy yielded 122 citations. After removing 25 duplicates, we screened the titles and abstracts of 98 articles. Furthermore, 24 articles were assessed for eligibility, and 16 studies were excluded that were not aligned with the pre-specified PICO (i.e., different intervention, wrong study design, and single-arm trial), resulting in eight RCTs [19-26] eligible for quantitative analysis. The PRISMA diagram for study selection is shown in Figure 1.

Studies Characteristics and Risk of Bias

Eight RCTs comprising 157 patients and 250 lesions were included in the final analysis. Erosive OLP type was reported in six RCTs, while the type of OLP in two studies was unknown [25, 26]. Five RCTs [22-26] were split-mouth approach, while the other three RCTs were case-control design. The duration of follow-ups ranged from 4 to 16 weeks of assessment; however, we considered only eight weeks as our last follow-up assessment. Three interventions were pooled: PRP (five RCTs), PRF (three RCTs), and corticosteroids (eight RCTs). Detailed baseline and summary are illustrated in Table 1.

**Table 1:** Baseline characteristics and summary data of the included studies. PRP - platelet-rich plasma; PRF - platelet-rich fibrin; NA - not available; RCT - randomized controlled trial; VAS - visual analog scale; NRS - numeric rating scale; OLP - oral lichen planus

Author, year	Country	Interventional modality	Study design	Patients, n	Age (years)	Female/male	OLP type	Pain assessment tools used	Therapy duration (months)	Follow up (months)
Ahuja et al., 2020 [19]	India	PRP	Case-control RCT	N=20, Interventional: 10, Corticosteroids: 10	44.5	18/2	Erosive	VAS	2	4
Hijazi et al., 2021 [21]	Egypt	PRP	Case-control RCT	N=20, Interventional: 10, Corticosteroids: 10	42.6 / 50.3	18/2	Erosive	VAS	1	3
Saglam et al., 2021 [23]	Turkey	PRF	Split-mouth RCT	N=24, Interventional: 24, Corticosteroids: 24	52.25	14/10	Erosive	VAS	2	6
Bennardo et al., 2021 [25]	Italy	PRF	Split-mouth RCT	N=9, Interventional: 9, Corticosteroids: 9	59.56 ± 3.57	6/3	NA	VAS	2	9
Al-Hallak et al., 2022 [22]	Syria	PRF	Split-mouth RCT	N=12, Interventional: 12, Corticosteroids: 12	48 ± 12.7	9/3	Plaque-like, ulcerative, atrophic, erosive	VAS	1	3
El Ghareeb et al., 2023 [20]	Egypt	PRP	Case-control RCT	N=24, Interventional: 12, Corticosteroids: 12	47 ± 13.12 / 52.17 ± 9.93	14/10	Erosive, reticular, mixed	NRS	2	3
Choudhary et al., 2023 [26]	India	PRP	Split-mouth RCT	N=28, Interventional: 28, Corticosteroids: 28	NA	NA	NA	VAS	1	2
Sharma et al., 2023 [24]	India	PRP	Split-mouth RCT	N=20, Interventional: 20, Corticosteroids: 20	35.75 ± 8.746	15/5	Erosive	VAS	2	4

**Figure 2:**
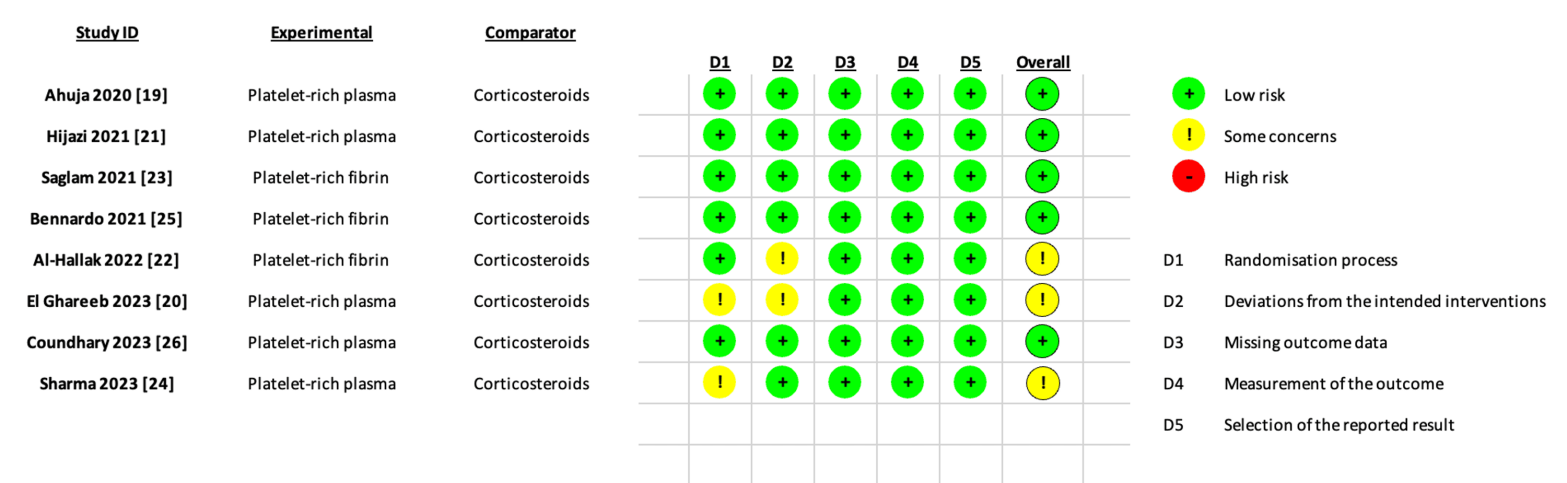
Risk of bias summary.

Regarding the ROB2 of RCTs, five studies were of low-risk bias, and three studies were of some concerns, mainly due to the lack of information on the randomization process and deviations from the intended interventions. Detailed ROB2 analysis is shown in Figure 2. Publication bias was assessed using funnel plots and was undetectable using the Egger tests (p>0.05).

Network Meta-Analysis: Efficacy Outcomes

Changes in lesion sizes: Data from three RCTs (n=57 patients, and 94 lesions) with two direct pairwise comparisons among three interventions were pooled at the first, second, third, fourth, and eighth weeks of follow-up. No significant differences were observed among the comparison. Moreover, the NMA reported that the three interventions may be similar to each other's in terms of changes of lesion size across different follow-up points. Summary estimates values are detailed in Table 2.

**Table 2:** League table of treatments for the assessment of lesion size, cm2 at different time points. Data presented as mean difference (95% confidence interval) PRP - platelet-rich plasma; PRF - platelet-rich fibrin Sources: [19-26]

Follow-up	Corticosteroids versus PRP	Corticosteroids versus PRF	PRP versus PRF
First week	0.39 (-0.62 to 1.40)	-0.30 (-1.59 to 0.99)	0.69 (-0.95 to 2.32)
Second week	0.10 (-0.80 to 1.01)	-0.47 (-1.78 to 0.84)	0.57 (-1.02 to 2.16)
Third week	-0.02 (-1.66 to 1.62)	-0.56 (-2.80 to 1.68)	0.54 (-2.24 to 3.31)
Fourth week	0.43 (-0.29 to 1.14)	-0.36 (-1.48 to 0.76)	0.79 (-0.54 to 2.12)
Eighth week	0.08 (-0.60 to 0.77)	-0.36 (-1.28 to 0.56)	0.44 (-0.71 to 1.59)

Compared to corticosteroids, NMA ranking suggested the best intervention was PRF (SUCRA values: 72.6%, 75.8%, 66.2%, 80.8%, and 77.5% for first, second, third, fourth, and eighth weeks of assessment), followed by PRP (SUCRA values: 55.3%, and 42.8% for first and third week of assessment), and SUCRA values of corticosteroids with 41.3%, 56.3%, and 39.7% at 2nd, 4th, and 8th weeks of assessment), as shown in Table 3.

**Table 3:** SUCRA values of treatments for the assessment of studied outcomes at different time points. Data presented as percentages (%) PRP - platelet-rich plasma; PRF - platelet-rich fibrin; SUCRA - surface under the cumulative ranking curve Sources: [19-26]

Outcomes	Treatment	SUCRA values				
		First week	Second week	Third week	Fourth week	Eighth week
Lesion size	PRP	55.3	32.9	42.8	12.8	32.8
	PRF	72.6	75.8	66.2	80.8	77.5
	Corticosteroids	22.1	41.3	41	56.3	39.7
Pain score	PRP	19.7	61.3	30.6	90.2	28.8
	PRF	91.8	52.3	86	20.4	85.9
	Corticosteroids	38.4	36.3	33.3	39.4	35.2
Thongprasom scores	PRP	--	--	--	41.7	78.1
	PRF	--	--	--	77.3	41.5
	Corticosteroids	--	--	--	30.9	30.3

Changes in pain scores: Data from six RCTs assessed the change in pain scores at the fourth and eighth weeks of follow-up, while only three, four, and five RCTs, respectively assessed the changes of pain scores at first, second, and third weeks of assessment. No significant differences were observed among comparison in the NMA model. The three interventions had similar pooled estimated compared to each other's in terms of changes of pain scores across all different time points of assessment. Detailed net leagues are summarized in Table 4.

**Table 4:** League table of treatments for the assessment of pain score at different time points. Data presented as mean difference (95% confidence interval) PRP - platelet-rich plasma; PRF - platelet-rich fibrin Sources: [19-26]

Follow-up	Corticosteroids versus PRP	Corticosteroids versus PRF	PRP versus PRF
First week	0.30 (-0.89 to 1.50)	-2.19 (-5.39 to 1.01)	2.49 (-0.92 to 5.91)
Second week	-0.28 (-1.38 to 0.81)	-0.20 (-2.17 to 1.77)	-0.08 (-2.33 to 2.17)
Third week	-0.00 (-1.00 to 1.00)	-1.64 (-4.64 to 1.36)	1.64 (-1.52 to 4.80)
Fourth week	-0.43 (-1.04 to 0.17)	0.24 (-0.79 to 1.26)	-0.67 (-1.85 to 0.51)
Eighth week	0.17 (-1.14 to 1.48)	-1.07 (-3.02 to 0.88)	1.24 (-1.15 to 3.63)

Compared to corticosteroids, NMA ranking suggested the best intervention was PRF at the first, third, and eighth weeks of assessment with the following SUCRA values, respectively (91.8%, 86%, and 85.9%), while PRP was the best intervention at second and 4th weeks of assessment with SUCRA values as follows (61.3%, and 90.2%), followed by corticosteroids with the SUCRA following values at first, second, third, fourth, and eighth weeks of assessment (38.4%, 36.3%, 33.3%, 39.4%, and 35.2%), as shown in Table 3.

Changes in Thongprasom-scale scores: Data from five RCTs assessed the changes in the Thongprasom-scale scores in the eighth week, while only three RCTs in the fourth week of follow-up. There were no significant differences observed among the interventions in terms of changes in Thongprasom-scale scores at the fourth or eighth weeks of assessment. Detailed net leagues are summarized in Table 5.

**Table 5:** League table of treatments for the assessment of Thongprasom score at different time points. Data presented as mean difference (95% confidence interval) PRP - platelet-rich plasma; PRF - platelet-rich fibrin Source: [19-26]

Follow-up	Corticosteroids versus PRP	Corticosteroids versus PRF	PRP versus PRF
Fourth week	-0.30 (-0.90 to 0.30)	-0.07 (-0.82 to 0.69)	-0.23 (-1.20 to 0.73)
Eigthth week	-0.02 (-0.47 to 0.43)	-0.26 (-0.79 to 0.27)	0.24 (-0.46 to 0.94)

Compared to corticosteroids, the NMA ranking suggested the best intervention was PRP in the fourth week of assessment with 78.1%, followed by PRF (SUCRA value: 41.5%) and corticosteroids (SUCRA value: 30.3%). At eight weeks of assessment, PRF was the best intervention was a SUCRA value of 77.3%, followed by PRP (SUCRA value: 41.7%) and corticosteroids (SUCRA value: 30.9%), as shown in Table 2.

Discussion

The current recommendations for OLP, including corticosteroids, are available to only relieve the symptoms and reduce the inflammation without a significant impact on the enhancement of patients' safety or their quality of life [27]. Our network meta-analysis systematically reviewed all the literature and included eight RCTs involving 157 patients and 250 lesions comparing PRF, PRP, and intralesional corticosteroids.

To the best of our knowledge, this is the first network meta-analysis to assess the effectiveness of autologous platelet concentrates compared to intralesional corticosteroids in patients with OLP. Our results showed that autologous platelet concentrates (PRP and PRF) were comparable to intralesional corticosteroids in terms of changes in lesion size and pain scores at the first, second, third, fourth, or eighth weeks of assessment. Additionally, they were comparable to corticosteroids to reduce the mean score of the Thongprasom Scale at the fourth and eighth weeks of assessment, without significant rates of adverse events.

Regarding lesion size, Ahuja et al. [19] and Bennardo et al. [25] reported a significant reduction in patients allocated to corticosteroids or autologous platelets concentrates at different follow-up intervals compared to the baseline; however, no significant differences were observed when compared to each other's. Furthermore, Ahuja et al. [19] reported that during the initial weeks, PRP showed slightly less reduction in lesion size, pain score, and Thongprasom scores compared to corticosteroids. However, during the later phase, PRP showed better or comparative results than corticosteroids, which could be explained by the difference between corticosteroids and PRP in the mechanism of action.

Lesion characteristics of OLP were documented to impair the functions of regulatory T lymphocyte, keratinocytes, and cell-matrix structure, and deficits in the cell growth factors as transforming growth factor β (TGF-β), and fibronectin [28]. The fact that PRP contains different growth factors, as platelet-derived growth factor (PDGF), epithelial growth factor (EGF), TGF-β, and fibronectin, all of which have a variety of functions as promotion of cell differentiation, proliferation, and regeneration [29]. Growth factors, as PDGF and TGF-β, specifically, have been documented to stimulate the proliferation of fibroblasts and collagen synthesis, with an additional enhancement of the expression of matrix metalloproteinases (MMPs), which regulate cell remodeling [30]. These properties might limit lesion extension in patients with OLP due to the nature of its cell proliferation and regeneration mechanism of action.

Regarding pain VAS score, Choudhary et al. [26], Ahuja et al. [19], ElGhareeb et al. [20], Hijazi et al. [21], and Sharma et al. [24] reported a significant reduction in both corticosteroids and PRP groups at different follow-up intervals compared to the baseline; however, no significant differences were observed when compared to each other's. Also, Bennardo et al. [25], Saglam et al. [23], and Al-Hallak et al. [22] reported comparable reduction in pain scores between corticosteroids and PRF groups; however, the corticosteroids group showed a nonsignificant reduction in pain scores compared to the PRF group. This could be explained by the anti-inflammatory response of corticosteroids on oral keratinocytes [31]. Also, the usage of a high concentration of intralesional corticosteroids every week in patients with OLP was associated with better results in reducing the lesion size, pain scores, and diminution of clinical scores by Thongprasom-scale due to the nature of the anti-inflammatory effect of corticosteroids and its ability on local regulation on T lymphocytes.

On the other hand, it is documented that PRF has an anti-inflammatory response by inhibiting the action of TLR4+ cells, which are primarily expressed in macrophages, dendritic cells, and some epithelial cells. These cells recognize lipopolysaccharides and trigger an inflammatory response. PRF inhibits this pathway by reducing the activation of TLR4 and blocking the classical inflammatory-related signaling cascade, specifically through the inhibition of p-p65 [32]. Considering the inflammatory mediators involved in OLP and the anti-inflammatory properties of PRF, it may serve as an effective alternative treatment option. This discussion strengthens the current literature, suggesting that both PRP and PRF could be promising therapeutic approaches for OLP.

Assessment of clinical scores by Thongprasom Score showed that no significant differences were observed at the fourth or eighth weeks of assessment between corticosteroids, PRP, and PRF. These findings were aligned with the previous literature by Ahuja et al. [19], ElGhareeb et al. [20], Hijazi et al. [21], Sharma et al. [24], Al-Hallak et al. [22], and Bennardo et al. [25]. The rate of recurrence and relapse, as well as the stability of the conditions play a great role in the severity of clinical scores. Ahuja et al. [19] reported only 1 of 10 patients allocated to the PRP group had a recurrence of symptoms, while 3 of 10 patients in the corticosteroids documented a recurrence during the eighth week of follow-up. Additionally, Al-Hallak et al. [22] reported a relapse rate with mild symptoms of 16.7% during the three-month follow-up in both groups, without significant difference, and Bennardo et al. [25] reported three of nine patients had mild relapse of symptoms on both buccal mucosa during follow-up.

On the contrary, Choudhary et al. [26] reported a significant reduction in clinical scores in patients treated with PRP as compared to intralesional corticosteroids. This could be explained by the usage of Kenacort 40mg as an intralesional steroid and the implantation of a different methodology of clinical assessment where they used the modified Escuider Index-10, which is different from the Thongprasom-scale used across the included studies. The major limitation of the Thongprasom Scale is it has not been validated, yet it has been widely used in the literature [33]. Also, it has low sensitivity because the scoring is more depicted to the clinical variant of the lesions rather than the assessment of real dimensional area.

Regarding the adverse events, Choudhary et al. [26], Sharma et al. [24], Hijazi et al. [21], and ElGhareeb et al. [20] showed similar rates in both groups. Despite the comparable estimate, ElGhareeb et al. [20] reported that among patients who received PRP, the frequency of pain was higher compared to intralesional corticosteroids. They also reported that patients treated with PRP was associated with a higher rate of remission compared to those treated with intralesional corticosteroids. Their results might be explained by the dilution of intralesional corticosteroids with lidocaine as a local anesthetic, which mitigates the local symptoms.

The emergence of PRP in different clinical settings was studied in patients undergoing tonsillectomy. Albazee et al. [34] included in their meta-analysis seven RCTs comprising 392 patients and reported a reduction in postoperative pain scores during the entire follow-up duration in the PRP group. Moreover, the incidence of postoperative hemorrhage either primary or secondary was reduced in the PRP group.

Studies reported different rates of remissions might be due to that patients with erosive OLP may show better results with PRP and PRF compared to other OLP types. Also, injection of PRP or PRF every week might enhance the healing process of erosive OLP due to the availability of sufficient growth factors required.

Moreover, our NMA showed that PRF was the best intervention with the highest SUCRA values compared to PRP and intralesional corticosteroids in all studied parameters. The regimen of PRF had major clinical advantages over PRP, such as of the use of tubes without anticoagulant, the smaller amount of blood used for preparation, the ease and speed of usage, and the lower costs of production. It also has a longer release of growth factors over time than PRP [35, 36].

Strengths and limitations

This NMA included only RCTs to ensure the reliability of the findings. Also, this is the first NMA to compare PRF, PRP, and intralesional corticosteroids. Furthermore, all of the included studies were of good quality. However, certain limitations associated with the included studies were found; there is a variety of doses and sessions in these studies. Also, due to the limited literature regarding the topic, the sample size is very low, which limits the generalizability of the results.

We recommend that patients diagnosed with OLP could have promising new lines of treatment, as PRF and PRP, and future multi-center RCTs with larger sample sizes and high quality are warranted to assess the optimal number of sessions, dosages, and specific endpoints. Also, global studies could be a good idea to measure the difference of prevalence, incidence and baseline severity of OLP in different regions population.

## Conclusions

PRF, PRP, and intralesional corticosteroids showed comparable results. There were no statistically significant differences in lesion size, pain scores evaluated by VAS or NRS, Thongprasom-scale score, and adverse effects. Also, this NMA strengthens the available literature on PRP and PRF, which could be potential and promising lines in the treatment of OLP. Future global studies measure the difference if prevalence, incidence, and baseline severity of OLP in different regions population, and multi-center three-arm RCTs with larger sample size and high quality maintaining the number of sessions, doses and specific endpoints are warranted.
